# Metagenomic evaluation, antimicrobial activities, and immune stimulation of probiotics from dietary supplements and dairy products

**DOI:** 10.1038/s41598-025-95664-w

**Published:** 2025-04-04

**Authors:** Piyaorn Chornchoem, Sarunporn Tandhavanant, Natnaree Saiprom, Anucha Preechanukul, Nartthawee Thongchompoo, Insee Sensorn, Wasun Chantratita, Narisara Chantratita

**Affiliations:** 1 Department of Microbiology and Immunology, Faculty of Tropical Medicine, Mahidol University, 420/6 Rajvithi Road, Bangkok, 10400 Thailand; 2 Division of Infection and Immunity, University College London, London, UK; 3 Center for Medical Genomics, Faculty of Medicine Ramathibodi Hospital, Mahidol University, Bangkok, Thailand; 4 Mahidol-Oxford Tropical Medicine Research Unit, Faculty of Tropical Medicine, Mahidol University, Bangkok, Thailand

**Keywords:** Probiotics, Antimicrobial activity, Metagenomic, Immune response, Supplements, Dairy products, Innate immunity, Microbiology, Microbial communities

## Abstract

Probiotics are widely marketed as dietary supplements and dairy products for their purported antimicrobial and immunomodulatory activities, often with limited supporting evidence. We identified and isolated probiotics from commercial dietary supplements and dairy products using metagenomics and cultured-based methods. We assessed their anti-bacterial activity against diverse pathogens and investigated their immunomodulatory effects on phagocytes and natural killer (NK) cells. Metagenomic analysis revealed that *Lactobacillus* and *Bifidobacterium* were the predominant genera in dietary supplements, while *Streptococcus* spp. was dominated in dairy products. However, only 37% of the predominant microorganisms identified by metagenomics were accurately listed on product labels. Among 70 representative probiotic strains, 4.3–17.1% probiotic strains demonstrated strong antibacterial-effects against pathogenic bacteria. Notably, specific strains of *Bifidobacterium longum* and *Lactobacillus plantarum* exhibited strong antagonistic activity against extended-spectrum beta-lactamase-producing and carbapenem-resistant *Escherichia coli*. Some strains of *Lactobacillus* spp. significantly enhanced phagocytic activity in monocytes and increased IFN-γ production in NK cells, while members of *Lactobacillus rhamnosus* significantly suppressed TNF-α, IL-6, and IL-8 production in lipopolysaccharide-stimulated macrophages. In contrast, *Bifidobacterium animalis* stimulated the production of anti-inflammatory cytokines. This study highlights discrepancies in probiotic labeling and demonstrates the antimicrobial and immunomodulatory potential of specific probiotic strains, suggesting their utility in enhancing health and wellness.

**Supplementary Information:**

The online version contains supplementary material available at 10.1038/s41598-025-95664-w.

## Introduction

Probiotics are live microorganisms, including bacteria and yeast, that promote health benefits to the host when consumed in adequate amounts. Lactic acid bacteria (LAB) are Gram-positive bacteria that produce lactic acid from carbohydrate fermentation, such as *Lactobacillus* spp., *Enterococcus* spp., *Bifidobacterium* spp. and *Saccharomyces* spp. These microorganisms are common probiotics formulated into dietary supplements and dairy products. Over 11,000 probiotics strains have been reported to exhibit biological activities that may benefit health^1^. The use of probiotics is increasingly recognized as a strategy to prevent infections and improve human and animal health^2–6^. The potential antimicrobial properties of probiotics and their products involve inhibiting the growth of pathogens^6–9^. In addition, probiotics can modulate immune responses, either activating^10–13^ or suppressing^14^ specific immune functions. These studies showed that probiotics interact with the host environment and influence the host immune response in various pathways.

Macrophages are a diverse group of immune cells with critical roles in both pro-inflammatory and anti-inflammatory responses. These cells help maintain immune balance and respond to infections and inflammation^15^. Different probiotic strains can influence cytokine production by macrophages, which can enhance or suppress inflammation. Due to the various effects of probiotic strains on immune activation, it is essential to evaluate the immunomodulatory properties of probiotics to ensure their safety and effectiveness in treating immune-related diseases^16^.

Natural killer (NK) cells are another important part of the innate immune system. These cells produce cytokines, such as interferon-gamma (IFN-γ), which activate other immune cells like macrophages and dendritic cells^17^. The secretion of IFN-γ is triggered by cytokines, microbial products, or direct interaction with infected or cancerous cells. Some strains of LAB have been shown to activate IFN-γ production by NK cells, suggesting their potential role in boosting immunity^18,19^.

Although many commercial probiotic products are available, studies using culture methods have shown inaccuracies in the labeling of their microbial compositions^20–22^. The mismatch between declared and actual probiotic species raises concerns about product reliability. Accurate methods, such as metagenomics, are needed to identify the whole-microbial genome present in these products and further verified by using standard culturing techniques to determine the viability of all the microbes^23^. Furthermore, manufacturers often claim that probiotics can restore gut health, support digestion, and modulate the immune system. However, the stability of probiotics can be affected when combined with other bacterial isolates, food ingredients, or supplements^24,25^.

Despite the potential health benefits of probiotics, little is known about how they interact with immune cells such as macrophages and NK cells. To address this gap, we hypothesized that metagenomics could be used to validate the composition of probiotics in commercial dietary supplements and dairy products. These probiotics might exhibit antimicrobial properties against harmful bacteria, including antibiotic-resistant pathogens, and influence cellular immune functions.

This study aimed to validate microbial species in commercially available dietary supplements and dairy products. We used a metagenomics approach to identify probiotics in 13 dietary supplements and 25 dairy products, as probiotics are commonly formulated into these products. The identified microorganisms were isolated and further analyzed for their antimicrobial properties. Additionally, we assessed their effects on cellular immune functions, including phagocytosis by monocytes, cytokine release by macrophages, and NK cell activation.

## Results

### Metagenomic detection of probiotics in dietary supplements and dairy products at the genus level

**Fig. 1:**
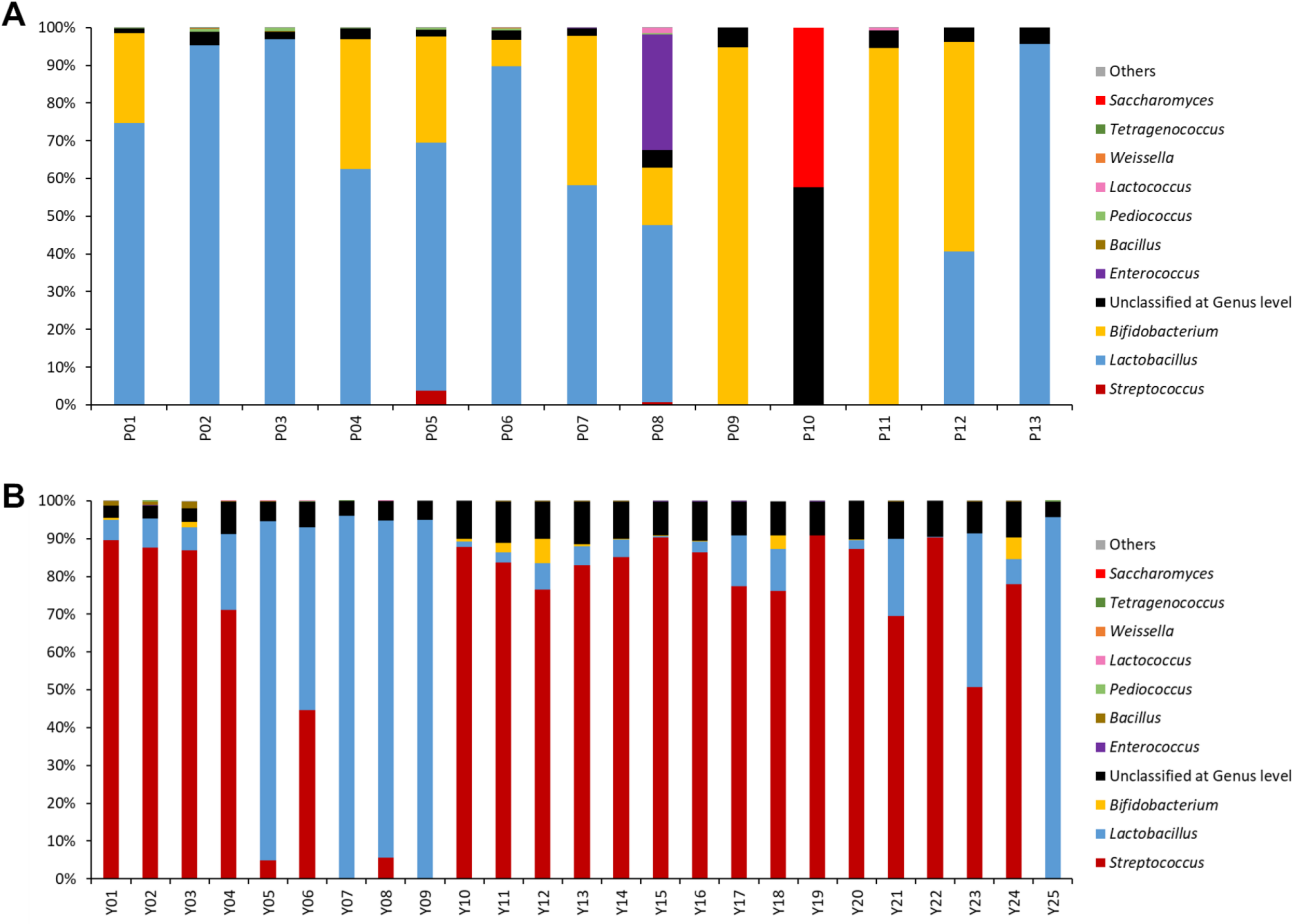
Metagenomic profiles of probiotics in dietary supplements (**A**) and dairy products (**B**).

**Fig. 2:**
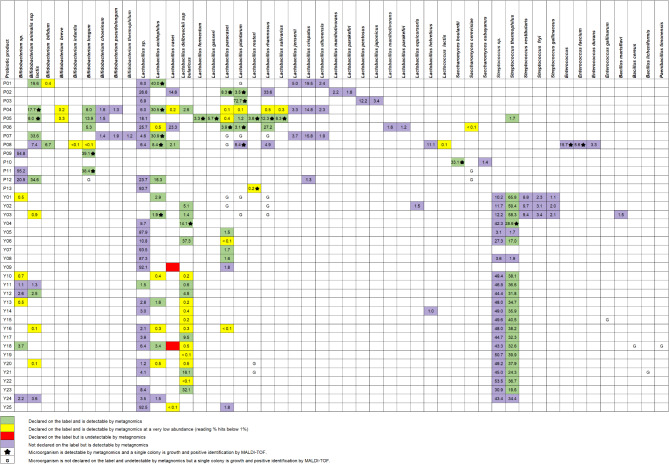
The comparison between the microorganism species declared on the labels of probiotic supplement products and those identified through metagenomic sequencing using bioinformatic analyses and cultivation methods. The comparison was performed from six perspectives. First, microbes present in the supplement that were declared on the label and detected through analysis (green). Second, microbes were declared on the label but detected at a very low abundance in the analysis (less than 1% of the reads) (yellow). Third, microbes were declared on the label but not detected via metagenomic analyses (red). Fourth, microbes were detected within the supplements’ metagenomic but were not declared on the label (purple). The numbers in the table indicated the % abundance of microbe in the metagenomic analysis. Fifth, microbes indicate with a star were detected in the supplement’s metagenomics and grown as a single colony and identified by MALDI-TOF. Finally, microbes indicate with a G were grown as a single colony and identified by MALDI-TOF but were not declared on the label and were undetectable by metagenomics.

A total of 13 dietary supplements (codes P01-P13) and 25 dairy products (codes Y01-Y25) were analyzed for the probiotic population using metagenomic analysis based on 16 S rRNA gene and ITS sequencing (Fig. 1). The composition of probiotics in each product, as indicated on the label, was compared with the results obtained from metagenomic analysis (Fig. 2, Supplementary Figure S1 and Supplementary Table S1). In this study, references to *Lactobacillus* at the genus level correspond to the broader lactobacilli group as classified in the GreenGenes database version 13.5, rather than the current taxonomic definition following the 2020 revision^26^.

Thirteen dietary supplements were indicated with 5 genera of microorganisms on their labels, including *Lactobacillus*, *Bifidobacterium*, *Saccharomyces*, *Lactococcus* and *Streptococcus*, which were discovered by metagenomic analysis (Figs. 1 and 2 and Supplementary Tables S1-2). Notably, the metagenomic analysis revealed additional genera not indicated on the product labels (Supplementary Figure S1 and Supplementary Tables S1- 2). For instance, *Enterococcus* was detected as the most abundant microorganism in dietary supplement P08 but was absent from the product’s label. Other low-abundance genera, such as *Acidomyces*, *Bifidobacterium*, *Lactobacillus*, *Lactococcus*, *Pediococcus*, *Plectosphaerella* and *Streptococcus*, were detected in dietary supplements P09-P13 without being listed on their labels.

Of the 25 dairy products, 24 indicated probiotics on their labels (Supplementary Tables S1). All 24 products (100%) revealed the presence of *Lactobacillus*, while *Streptococcus* and *Bifidobacterium* were displayed on 19 (76%) and 8 (32%) dairy products, respectively. Metagenomic analysis confirmed the presence of all probiotics indicated on the labels of these products, but in a lower proportion than stated. In addition, a small amount of other bacterial genera not indicated on the labels, such as *Bacillus*, *Bifidobacterium*, *Pediococcus*, *Streptococcus* and *Tetragenococcus*, were detected by metagenomic analysis in several dairy products (Y01-09, Y12, Y14, Y16, Y22, Y23 and Y25).

### Metagenomic detection of probiotics in dietary supplements and dairy products at species level

Seventeen species of probiotics were listed on the labels of 13 dietary supplements, including 10 species of *Lactobacillus*, 4 species of *Bifidobacterium*, and 1 species each of *Lactococcus*, *Streptococcus* and *Saccharomyces* (Supplementary Table S1). Metagenomic analysis at the species level revealed all indicated probiotics on product labels with lower relative abundance (Figs. 1 and 2, Supplementary Figure S2 and Supplementary Tables S1-2). Resemble with the genus classification, *Enterococcus faecium*, unlisted on the labels, was found in P08.

The dairy products had less diversity of probiotics than dietary supplements. Only 6 species of probiotics were commonly listed in 24 dairy products: *Bifidobacterium animallis* subsp. *lactis*, *Lactobacillus acidophilus*, *Lactobacillus delbrueckii* subsp. *bulgaricus*, *Lactobacillus casei*, *Lactobacillus paracasei* and *Streptococcus salivarius* subsp. *thermophilus*. Almost indicated probiotics on product labels were detected by metagenomic analysis (Figs. 1 and 2, Supplementary Figure S2 and Supplementary Tables S1-2). Only *L. casei*, was not classified in products Y09 and Y18.

The top ten microorganisms identified by metagenomic analysis of all products are shown in Supplementary Table S2. The median proportion of probiotics in the top ten list was 95.03% (range 52.5–97.65, IQR 94.2–95.6%) of the DNA library. Only 38.6% of microbes in the top ten list from the metagenomic analysis matched with those indicated on the product labels.

Misidentification of probiotics at the species level was more prevalent in dietary supplements (abundance range: 1.5 − 54%) compared to dairy products (abundance range: 0.81 − 18.3%). These findings highlight discrepancies between label information and the actual microbial composition.

### Isolation of probiotics from supplemental food products

All 13 dietary supplements and randomly selected 8 of 25 dairy products were cultured and isolated for probiotics (Supplementary Tables S1-3). From these 21 samples, 671 colonies were isolated, and 22 microbial species were identified by MALDI-TOF analysis. Among these, 13 species matched the probiotic listed on the labels of 11 dietary supplements and 4 dairy products. These species included *B. animalis* (2 products), *B. longum* (2 products), *L. acidophilus* (5 products), *L. bulgaricus* (1 product), *L. fermentum* (1 product), *L. gasseri* (1 product), *L. paracasei* (4 products), *L. plantarum* (4 products), *L. reuteri* (2 products), *L. rhamnosus* (1 product), *L. salivarius* (2 products), *S. thermophilus* (3 products) and *S. cerevisiae* (1 products). However, 4 species (*B. bifidum*,* B. breve*,* L. casei* and *L. lactis)* were initially isolated but could not be maintained in culturing conditions used in this study. We were unable to isolate the probiotics listed on the labels of 2 dietary supplements (P08 and P12) and 4 dairy products (Y01, Y02, Y15 and Y21). In addition, 5 species were isolated but not listed on the label of any products, including *Bacillus cereus* and *Paenibacillus timonensis* from Y18, *Bacillus licheniformis* from Y21, *Enterococcus faecium* from P08 and *Enterococcus gallinarum* from Y15.

Many species such as *L. plantarum*,* L. paracasei*,* L. rhamnosus*,* L. reuteri*,* B. longum* were isolated by culture of 10 products (P01, P07, P08, P11, P12, Y01, Y02, Y03, Y20 and Y21) but were not indicated on their labels. These microorganisms were also detected at very low abundance by metagenomic analysis.

From 671 isolates, 70 probiotic strains were randomly selected and used as representative strains to evaluate anti-bacterial and immunomodulation activities. For each species, one isolate per product was selected, and 3 isolates per species were included when multiple strains of the same microorganism were labeled on a product. The selected isolates included *B. animalis* (N = 12), *B. longum* (N = 3), *E. faecium* (N = 1), *E. gallinarum* (N = 1), *L. acidophilus* (N = 10), *L. delbruckii* (N = 1), *L. fermentum* (N = 1), *L*,* gasseri* (N = 1), *L. paracasei* (N = 8), *L. plantarum* (N = 9), *L. rhamosus* (N = 13), *L. reuteri* (N = 2), *L. salivarius* (N = 2), *S. thermophilus* (N = 3) and *S. cerevisiae* (N = 3).

### Antimicrobial activity of probiotic isolates against pathogenic bacteria

**Fig. 3:**
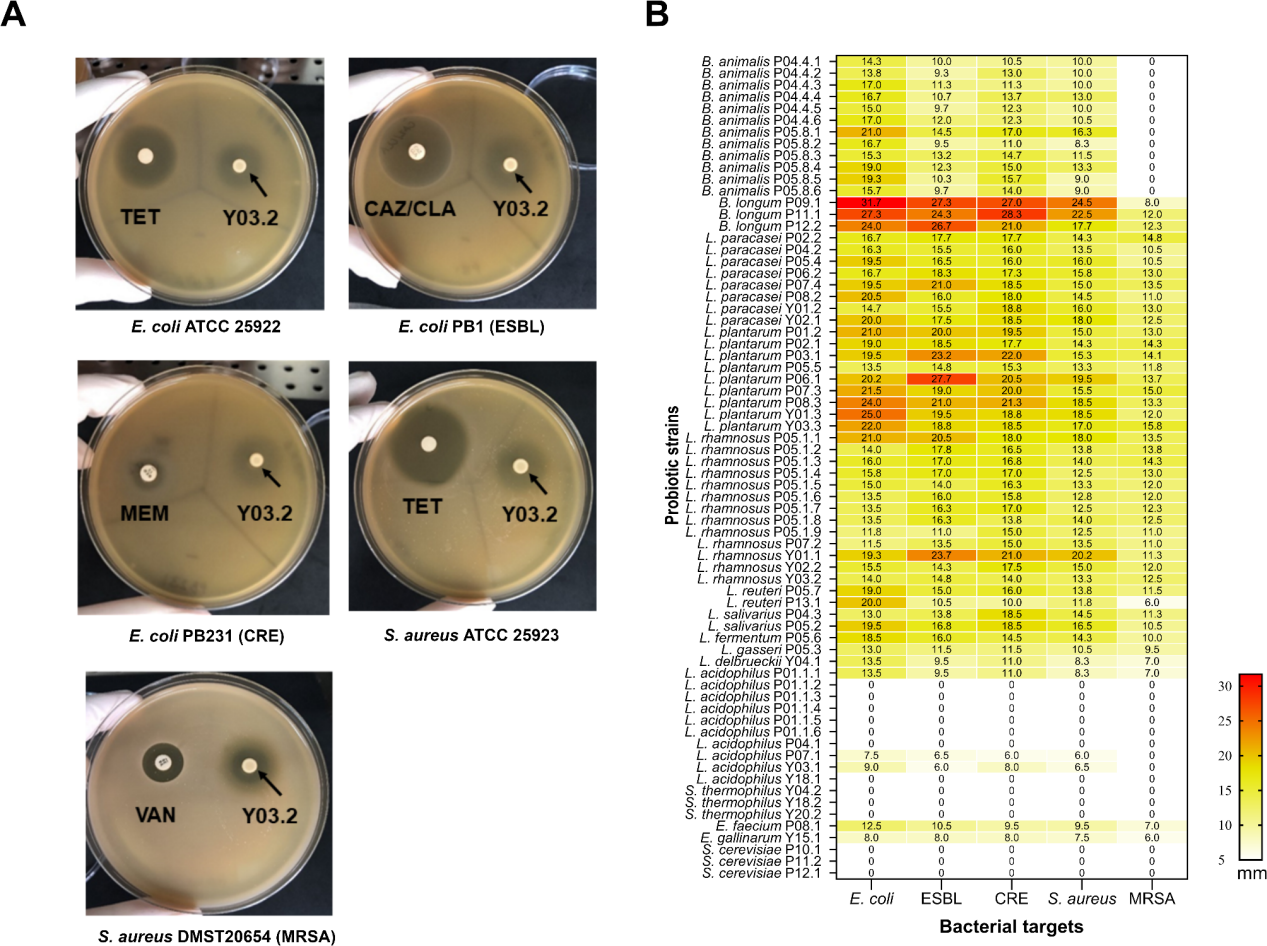
Antibacterial activity of probiotic isolates against antibiotic resistance bacteria by agar overlay method. (A) Agar overlay method demonstrated the antibacterial activity of live *L. rhamnosus* Y03.2 against five bacterial pathogens used as target strains, including *E. coli* ATCC25922, *E. coli* PB1 (ESBL), *E. coli* PB231 (CRE), *S. aureus* ATCC25923 and *S. aureus* DMST20654 (MRSA). The inhibition zones were observed surrounding probiotic spots (arrows). The diameter of the inhibition zone (mm), including probiotic spots (approximately 5 mm in diameter) was measured, and reported as means of two independent experiments. (B) The zone of inhibition of 70 tested probiotic strains was summarized in a heatmap chart. The diameter of the inhibition zone was interpreted as follows: >20 mm as a strong activity (red), 10–20 mm as a moderate activity (yellow), and < 10 mm as a weak inhibition (bright yellow).

Anti-bacterial activity of 70 live probiotic isolates against five pathogenic bacteria was evaluated using the agar overlay method (Fig. 3A). The diameters of inhibition zones were summarized in a heatmap chart (Fig. 3B) and categorized as follows: strong activity, inhibition zone > 20 mm; moderate activity, inhibition zones 10–20 mm; and weak activity, inhibition zones < 10 mm. The anti-bacterial activities varied depending on the probiotic species and strains. 51.4–64.3% of isolates demonstrated inhibitory effects with moderate levels against pathogenic bacteria. However, the overall effectiveness of the probiotics was lower against Methicillin-resistant *Staphylococcus aureus* (MRSA). (Table 1). Interestingly, 12.9% and 10.0% of probiotic isolates showed strong antimicrobial activity, with inhibition zone > 20 mm against antibiotic resistant bacteria including *E. coli* PB1 (Extended-spectrum β-lactamase; ESBL) and *E. coli* PB231 (Carbapenem-resistant Enterobacterales; CRE). Strong antimicrobial activity was particularly observed in isolates of *B. longum* (P09.1, P11.1 and P12.2), *L. plantarum* (P03.1, P06.1 and P08.3) and *L. rhamnosus* (Y01.1). In contrast, species such as *L. acidophilus*, *S. cerevisae* and *S. thermophillus* showed no anti-bacterial activity against any of the target bacteria (Fig. 3B). These findings suggest that certain probiotic strains, particularly *B. longum*,* L. plantarum*,* and L. rhamnosus*, have promising antimicrobial potential against multidrug-resistant bacteria, whereas others may lack anti-bacterial efficacy.

**Table 1:** The number of isolated probiotics showed antibacterial effects against tested organisms.

Level	Number of isolated probiotics showed antibacterial effects against the tested organisms (% of probiotics)				
	*E. coli* ATCC25922	*E. coli* PB1 (ESBL)	*E. coli* PB231 (CRE)	*S. aureus* ATCC25923	*S. aureus* DMST20654 (MRSA)
Strong antibacterial activity (> 20 mm)	12 (17.1%)	9 (12.9%)	7 (10.0%)	3 (4.3%)	0 (0.0%)
Moderate antibacterial activity (10–20 mm)	41(58.6%)	39 (55.7%)	45 (64.3%)	45 (64.3%)	36 (51.4%)
Weak antibacterial activity (< 10 mm)	17 (24.3%)	22 (31.4%)	18 (25.7%)	22(31.4%)	34 (48.6%)

### Effect of probiotics on phagocytosis enhancement in THP-1 monocytes

**Fig. 4:**
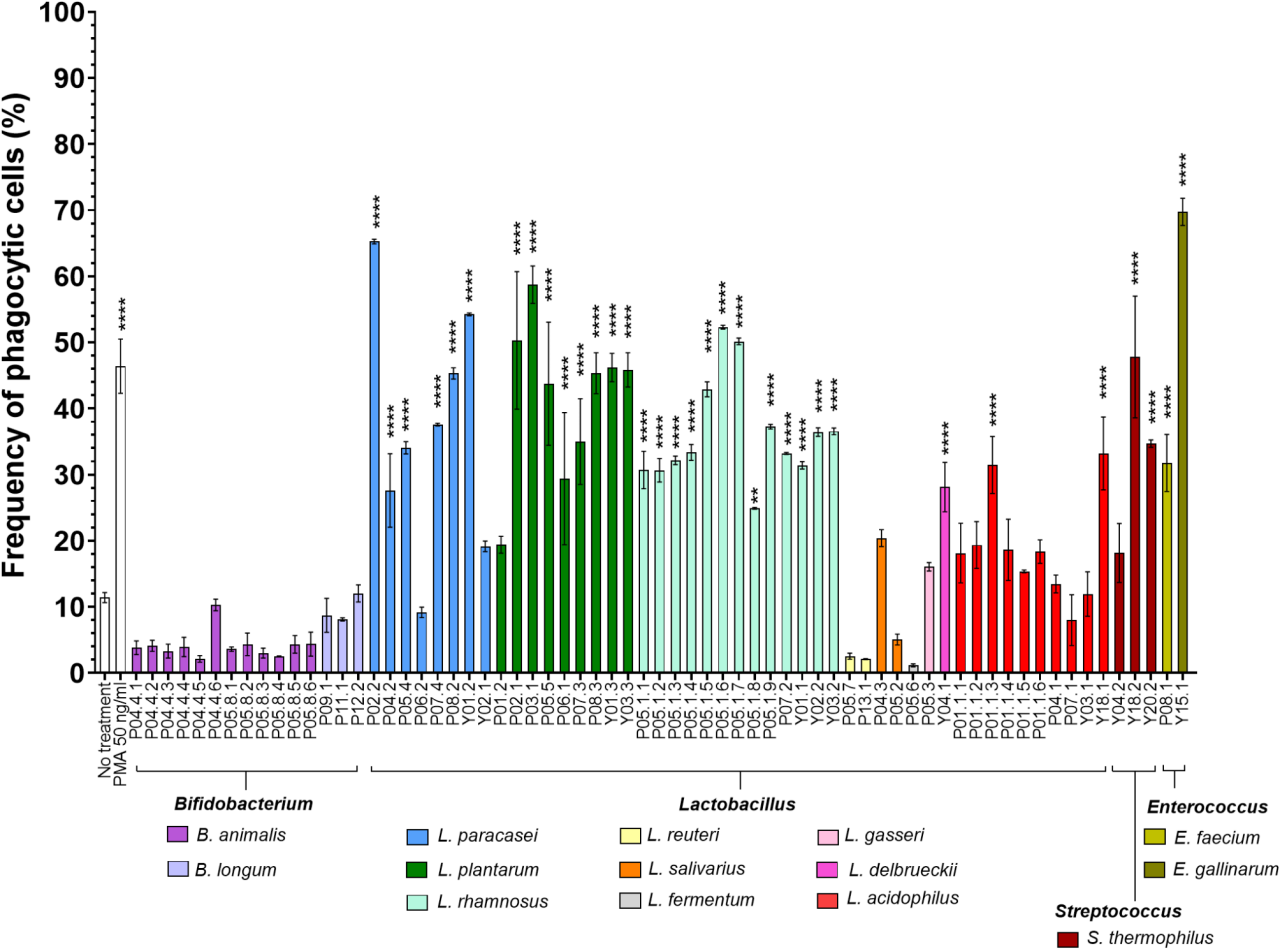
Effect of probiotics on phagocytic activity. The phagocytic index is defined as % phagocytosis of monocyte cells × mean fluorescence intensity. Monocyte with RPMI medium was used as the negative control (No treatment), and monocyte treated with PMA was the positive control. Each color indicates probiotic species. Data were presented as bar graphs with mean ± standard deviation from three independent experiments. One-way ANOVA was used to test the difference between probiotic bacteria affecting phagocytic activity. (****, *P* < 0.0001, ***, *p* < 0.001, **, *p* < 0.01).

Several dietary supplements claimed immune-supporting benefit (Supplementary table S4). To evaluate these claims, we determined the effect of probiotics on phagocytosis activity in THP-1 monocyte cells (Fig. 4). THP-1 cells were stimulated with probiotics at an MOI of 200, and their phagocytic activity was assessed using heat-killed, CFSE stained *E. coli*. Interestingly, stimulation of THP-1 cells with all tested strains of *L*. *rhamnosus* and *Enterococcus* spp. significantly increased phagocytosis of heat-killed *E. coli* compared to untreated cells (*p* < 0.0001). However, the enhancement of phagocytosis was strain dependent for *L. paracasei*,* L. plantarum*,* L. salivarius* and *L. acidophilus*. Moreover, the pre-treatment with *Bifidobacterium* spp., *L. reuteri*, *L. fermentum*, and *S. thermophilus* suppressed the phagocytosis activity of THP-1 cells. These findings indicate that certain probiotic species, particularly *L. rhamnosus*, and *Enterococcus* spp., may enhance innate immune functions, while others, such as *Bifidobacterium* spp. and *L. reuteri*, may have an inhibitory effect on monocyte phagocytosis.

### Effect of probiotics on cytokine production from THP-1-derived macrophages stimulated with LPS

**Fig. 5:**
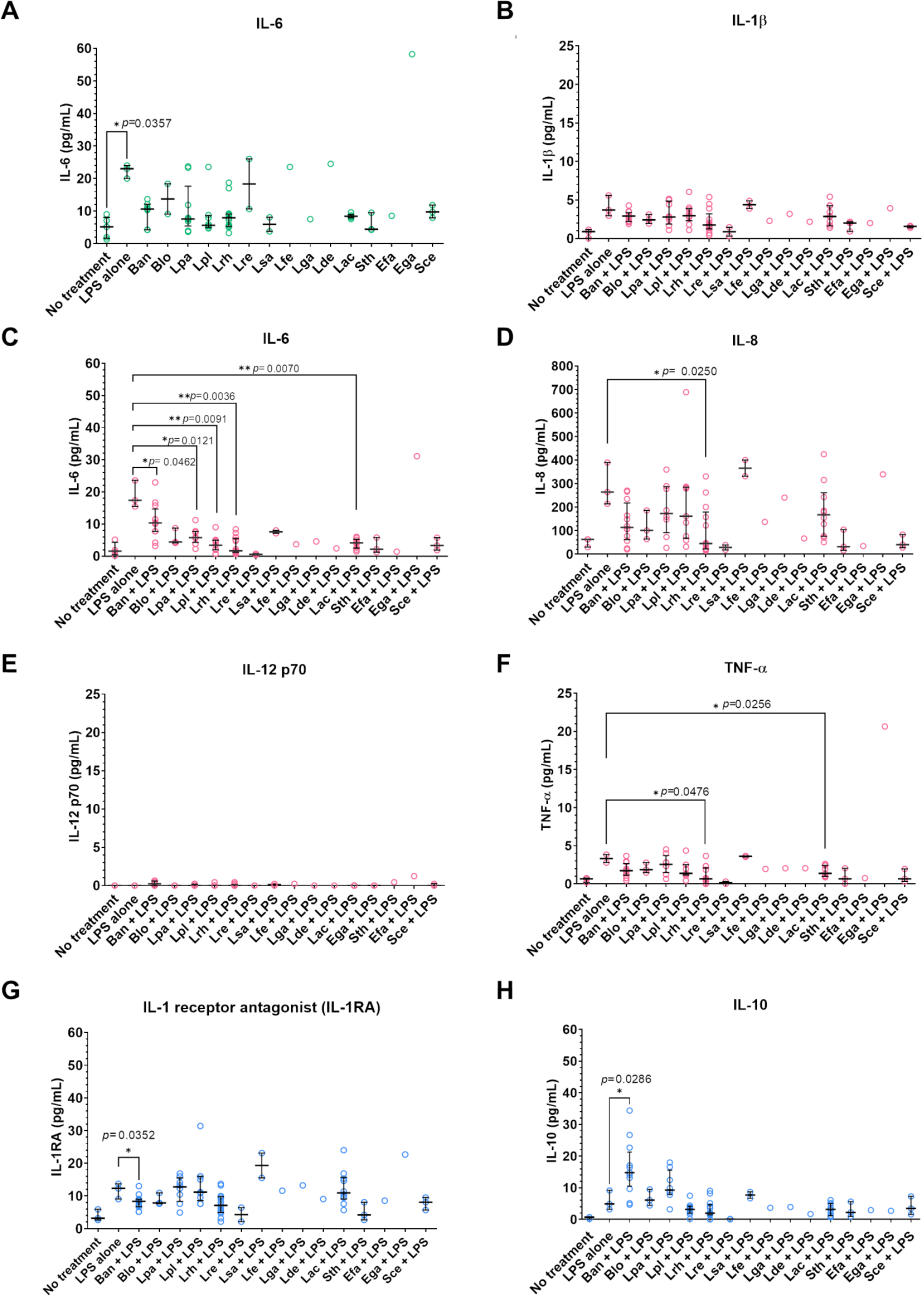
Effect of probiotics on cytokine production. Effect of probiotics on cytokine profiles from THP-1-derived macrophages or stimulated with LPS 1 µg/ml for 18 h. The supernatant was collected to measure cytokine profiles using Luminex assay. Cells with RPMI medium was used as a negative control and cells treated with LPS 1 µg/ml alone was used as a positive control. (**A**) IL-6 production from THP-1-derived macrophages after stimulating with probiotic strains. (**B**–**F**) IL-6, IL-8, IL-12p70 and TNF-α levels from cells treated with LPS and probiotic strains for 18 h. (**G**–**H**) IL-1RA and IL-10 from cells treated with LPS and probiotic strains for 18 h. (Mann-Whitney test; *, *p* < 0.05, **p < 0.01). (Ban; *B. animalis*, Blo; *B. longum*, Lpa; *L. paracacei*, *L. plantarum*, Lrh; *L. rhamnosus*, Lre; *L. reuteri*, Lsa; *L. salivarius*, Lfe; *L. fermentum*, Lga; *L. gasseri*, Lde; *L. delbrueckii*, Lac; *L. acidophilus*, Sth; *S. thermophilus*, Efa; *E. faecium*, Ega; *E. gallinarum*, Sce; *Saccharomyces cerevisiae*)

To evaluate the immunomodulatory effects of probiotics, we examined the impact on cytokine production in THP-1-derived macrophages. The pro-inflammatory cytokine, IL-6 was determined following stimulation with 1000 ng/ml of LPS as a positive control. Stimulation with LPS alone significantly increased IL-6 levels in THP-1 derived macrophage. However, treatment with probiotics without LPS showed no significant difference in IL-6 production compared to untreated cells (RPMI medium), indicating that these probiotics do not induce inflammation via IL-6 production. (Fig. 5A).

Next, we investigate whether probiotics isolated from dietary supplement and yogurt products could modulate the inflammatory response induced by LPS. THP-1-derived macrophages were co-treated with LPS and probiotics, and levels of pro-inflammatory cytokines (IL-1β, IL-6, IL-8, IL-12p70, and TNF-α) as well as anti-inflammatory cytokines (IL-1RA and IL-10) were measured (Fig. 5B-H). Stimulation with LPS alone led to elevated levels of IL-6, IL-8 and TNF-α and IL-1RA and IL-10 compared with untreated cells. However, co-treatment with probiotics significantly altered the cytokine response. For example, IL-6, IL-8 and TNF- α productions were decreased when co-stimulated with *L. rhamnosus* while IL-10 production was enhanced especially when co-stimulated with *B. animalis*. These findings suggest that while the probiotics tested do not directly induce inflammation, they can attenuate LPS-induced inflammatory responses in THP-1-derived macrophages by reducing pro-inflammatory cytokines and promoting anti-inflammatory cytokines.

### Enhancement of NK cell activity by probiotics

**Fig. 6:**
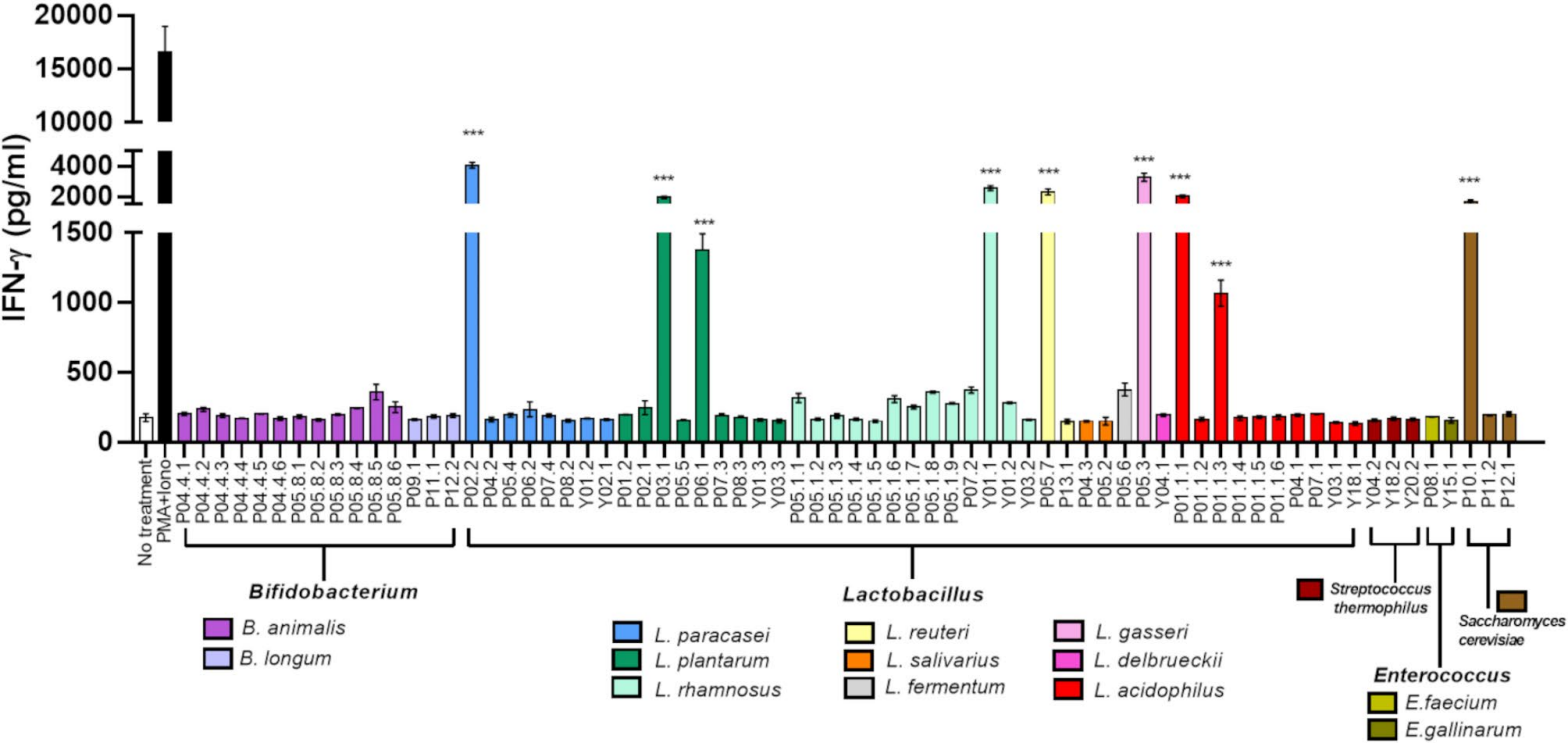
IFN-γ production by NK-92 MI cells activated with different probiotics. NK-92 MI cells were incubated with 70 isolates of probiotic bacteria from 16 species for 24 h. Mixture of PMA and ionomycin was a positive control and medium was a negative control. The supernatant was collected to measure IFN-γ concentration using ELISA assay. Data were represented as a bar graph with mean ± standard deviation from three independent experiments. One-way ANOVA was used to test the difference between probiotic bacteria affecting NK cell-derived IFN-γ production. ***, *p* < 0.0001.

To assess the impact of probiotics on innate immune response, we evaluated their ability to activate NK cells. NK-92 MI cell lines were co-cultured with 70 probiotic isolates from 16 species for 24 h, and the levels of IFN- γ in the supernatant were measured (Fig. 6). Most probiotic strains tested did not elicit NK-92 MI cells responses. However, 9 isolates of probiotics strains were able to stimulate NK-92 MI cells to secrete low levels of IFN-γ compared to the positive control. These isolates included *L. paracasei* (P02.2), *L. plantarum* (P03.1 and P06.1), *L. rhamnosus* (Y01.1), *L. reuteri* (P05.7), *L. gasseri* (P05.3), *L. acidophilus* (P01.1.1 and P01.1.3) and *S. cerevisae* (P10.1). The results suggest potential strain-specific immunomodulatory effects of probiotics on NK cells.

## Discussion

Dietary supplements and dairy products that contain probiotics frequently claim the health advantages of probiotics for promoting consumption. This study evaluated the microbial composition, antimicrobial activity, and immunomodulatory effects of probiotics from dietary supplements and dairy products using a metagenomics approach, culture-based isolation, and immune function assays. The findings highlight discrepancies between product labels and actual microbial content, provide insights into the antimicrobial properties of specific probiotic strains, and explore their potential to modulate innate immune responses.

Our metagenomic analysis revealed notable discrepancies between the labeled and actual microbial compositions of probiotics in both dietary supplements and dairy products. While the labels accurately listed some genera and species, many products contained additional bacterial genera, including *Enterococcus* and *Bacillus*, not mentioned on the labels. Furthermore, some strains listed on the labels, such as *L. casei*, were not detected in specific products. In addition, the culture method was able to isolate *E. faecium* from dietary supplement P08, suggesting contamination. These findings align with previous reports of inconsistencies in probiotic labeling^20,21^ and emphasize the importance of rigorous quality control and accurate product labeling to ensure consumer trust and product efficacy.

The success of isolating probiotics from dietary supplements and dairy products is significantly influenced by the composition of the culture medium. In this study, only 29 of the 70 microbes indicated on the label of 21 products were successfully isolated. Media such as MRSc and M17, commonly used for the cultivation of lactic acid bacteria were insufficient to promote the growth of all probiotics in each product. This limitation aligns with previous studies that suggest the need to optimize media compositions to support the growth of diverse lactic acid bacteria^27^. Specific nutrients or supplements, such as carbon sources, vitamins, or growth factors may be required to enhance the recovery of certain strains. Furthermore, the blending of several probiotics in a single product can further complicate isolation. Competitive interactions among co-cultured strains may suppress the growth of less dominant species, particularly in nutrient-limited media^28^. It is possible that the probiotic strains may enter a viable but non-culturable (VBNC) state during production or storage, rendering them difficult to isolate under conventional conditions. Resuscitation of VBNC cells often requires specific environmental conditions, such as changes in temperature, pH, or nutrient availability, which are not provided by standard media^28^. This highlights the need for advanced culturing techniques to recover these dormant but viable microorganisms^28^.

The antimicrobial activity of probiotics varies significantly by species and strains. Over half of the isolates exhibited moderate inhibitory effects against a panel of pathogenic bacteria, with strong activity observed in *B. longum*, *L. plantarum*, and *L. rhamnosus* against antibiotic-resistant strains such as *E. coli* PB1 (ESBL) and *E. coli* PB231 (CRE). In contrast, species like *L. acidophilus*, *S. cerevisiae*, and *S. thermophilus* showed no anti-bacterial activity. These results highlight the strain-specific nature of antimicrobial properties and suggest that certain strains may serve as promising candidates for combating multidrug-resistant pathogens.

LAB, particularly lactobacilli, have demonstrated the ability to combat bacterial pathogens through various mechanisms. These include producing antimicrobial substances such as hydrogen peroxide, organic acids (mainly lactic acid), and bacteriocins^29^, interacting with resident microbiota and host, improving gut barrier integrity, and producing enzymes^30^. Consistent with previous studies, our results revealed that *B. longum*, *L. plantarum*,* L. rhamnosus*, and *L. reuteri* strains exhibited remarkable antagonistic activity against *S. aureus* ATCC25923 and *E. coli* ATCC25922, with the inhibition profile varying by strains^31^. Furthermore, LAB probiotic strains in our study, especially *B. longum* and *L. plantarum* exhibited strongly antagonistic activity against ESBL- and CRE-*E. coli*. Previous study supports these findings, demonstrating that the cell-free supernatant (CFS) of *L. plantarum*,* L. rhamnosus* and *L. paracacei* strains exhibit significant anti-CRE activity using agar well diffusion and time-kill assay^32^. Additionally, a recent study revealed that CFS of *B. longum* FB1-1 inhibits the transfer of drug resistance plasmids among carbapenem-resistant *Klebsiella pneumoniae* strains^33^. While in vitro findings may not directly translate to in vivo outcomes, our study suggests that specific *Lactobacillus* and *Bifidobacterium* strains hold significant potential for preventing colonization by ESBL and CRE pathogens. Nonetheless, further studies, particularly in animal models, are needed to confirm these promising findings and explore their clinical relevance.

The innate immune system is essential for defending the host against pathogens. Key immune cells in the innate immune response include macrophages, dendritic cells, and natural killer (NK) cells. Several researchers have suggested that intestinal homeostasis can be restored through supplements containing fermented dairy products and beneficial microorganisms, such as *Lactobacillus* probiotics. For example, a previous study demonstrated that healthy adults consuming 300 g/day of yogurt supplement containing *L. acidophilus* 74 − 2 and *B. lactis* 420 showed a significant increase in granulocytes and monocytes phagocytic activity during the probiotic intervention^34^. Our study demonstrated diverse effects of probiotics on monocyte phagocytic activity. *L. rhamnosus*, *E. faecium* and *E. gallinarum* significantly enhanced phagocytosis, suggesting a role in boosting innate immune defense. Additionally, *L. paracasei*,* L. plantarum*,* L. salivarius* and *L. acidophilus* were strains dependent on enhancing phagocytosis. In contrast, *Bifidobacterium* spp. and *L. reuteri* suppressed phagocytosis. These findings underscore the need to evaluate strain-specific immunomodulatory effects, as different probiotics may have opposing effects on innate immune functions.

A previous study reported that heat-killed *L. plantarum* KCTC 13314BP enhances the phagocytic activity of RAW264.7 macrophages *via* activation of MAPK and STAT3 pathways^35^. Similarly, our results suggest that strains of *L. plantarum*, *L. rhamnosus* from dietary supplements and yogurt products may enhance phagocytic activity and have the potential to protect the host from pathogens.

Macrophages play a key role in maintaining intestinal homeostasis as immune sentinels in the gastrointestinal tract. They infiltrate the lamina propria and release cytokines to regulate and activate the innate immune responses. During infections, monocyte-derived macrophages migrate into the intestine, triggering an inflammatory response. Disruptions in macrophage balance contribute to immune damage in chronic inflammatory diseases^36,37^. In our study, Gram-positive probiotics were shown not to induce IL-6 production, indicating their minimal pro-inflammatory effect. Conversely, *E. coli*-LPS activated toll-like receptor 4 (TLR4), leading to the secretion of pro-inflammatory cytokines such as TNF-α, IL-1β, IL-6, and IL-8. These cytokines are crucial for the host’s defense against bacterial colonization and invasion^38^. Additionally, TLR5 recognizes flagellin from both Gram-negative and Gram-positive bacteria, activating the NF-κB pathway to further amplify pro-inflammatory responses^39^.

Our results revealed that LPS-induced TNF-α, IL-6, and IL-8 secretion by THP-1-derived macrophages was significantly reduced by probiotic strains, particularly *L. rhamnosus*, which suppressed all three cytokines. *L. acidophilus* strains suppressed TNF-α and IL-6, while *B. animalis*,* L. paracasei*, and *L. plantarum* selectively suppressed IL-6. These effects are likely to reflect strain-specific alterations in TLR4 expression, as observed in previous studies^40^.

In addition to reducing pro-inflammatory cytokines, *B. animalis* significantly induced the production of anti-inflammatory cytokines such as IL-10 and IL-1RA in LPS-stimulated macrophages. Intestinal macrophages consistently produce IL-10, which plays a critical role in maintaining immune tolerance by regulating regulatory T cells (Tregs). This interplay between macrophages and Tregs, both major sources of IL-10, is essential for balancing immune responses and preventing excessive inflammation in the intestinal mucosa^36^.

Natural killer (NK) cells play a critical role in combating infections and cancer. They destroy infected or abnormal cells by releasing cytolytic granules, such as perforin and granzymes, and secrete cytokines like IFN-γ and TNF-α, which regulate the immune cells. Probiotics have shown the potential to enhance NK cell activity. For instance, *L. casei* Shirota (LcS) has been demonstrated to boost NK cell activity in vitro by increasing the expression of surface activation markers CD69 and CD25 on CD8^+^ and CD56^+^ subsets of peripheral blood mononuclear cells (PBMCs), even without additional stimuli^41^. Similarly, the consumption of dairy yogurt containing *L. paracasei* ssp. paracasei, *B. animalis* ssp. lactis, and heat-treated *L. plantarum* has been shown to enhance immune function in immunocompromised elderly individuals by increasing NK cell activity and the production of IL-12 and IFN-γ^42^.

In our study, certain probiotic strains directly stimulated NK cells to secrete higher levels of IFN-γ compared to untreated controls. These strains included *L. paracasei* P02.2, *L. plantarum* P03.1, *L. plantarum* P06.1, *L. rhamnosus* Y01.1, *L. reuteri* P05.7, *L. gasseri* P05.3, *L. acidophilus* P01.1.1 and P01.1.3, as well as the yeast *S. cerevisiae* P10.1. Given the critical role of IFN-γ in enhancing macrophage and NK cell activity, this cytokine likely contributes to the anticarcinogenic and anti-infectious effects of these probiotics.

This study highlighted significant strain-specific differences in antimicrobial and immunomodulatory effects. However, there are several limitations. First, the analysis was limited to a subset of strains isolated from the products. It is possible that other strains present in low abundance or VBNC state were not captured, potentially underestimating the full scope of probiotic diversity and functionality. Second, conventional culture methods, such as MRSc and M17 media, were insufficient to isolate all the probiotics listed on product labels. The inability to cultivate certain strains or resuscitate VBNC cells limits the comprehensiveness of the analysis and highlights the need for optimized or alternative culturing techniques. Third, the antimicrobial and immunomodulatory activities of probiotics were evaluated using in vitro models, which do not fully replicate the complexity of the human gut environment. The observed effects may not directly translate to in vivo outcomes due to differences in microbiota interactions, immune system dynamics, and environmental conditions. Fourth, while metagenomics provided detailed insights into microbial composition, the resolution was limited at the strain level for some genera. This may have affected the accuracy of identifying specific strains and their relative abundances. Lastly, variability in manufacturing processes, storage conditions, and product formulations may have influenced the viability and functionality of probiotics. These factors were not systematically controlled or analyzed, potentially affecting reproducibility.

In conclusion, our study demonstrated significant discrepancies between product labels and actual microbial content, emphasizing the need for quality control and accurate labeling to ensure product efficacy and consumer trust. However, the accuracy and reliability of metagenomic studies in dietary supplements and dairy products depend on multiple factors. Metagenomic sequencing alone is unable to differentiate viable and non-viable microbes in samples. Many probiotic strains are not well-represented in public databases, leading to misclassification or incomplete identification. The algorithm used for DNA analysis may have limitations in distinguishing between closely related species. In multi-strain products, some probiotics may overcome others during culture on medium, leading to dominance in culture-dependent identification. The antimicrobial activity of probiotics varied by strain. These findings highlight the potential of specific probiotics in combating multidrug-resistant pathogens through different mechanisms. In addition, probiotics exhibited diverse effects on immune modulation, and we demonstrated that several strains directly stimulated NK cells to secrete higher levels of IFN-γ. Overall, this study underscores the strain-specific nature of probiotics and their potential to support antimicrobial and immunomodulatory therapies. Further research is needed, particularly in vivo studies, to validate these findings and explore their clinical applications in infection control and immune health.

## Methods

### Probiotic products

Thirteen dietary supplements (referred to as product code P01-P13) and 25 dairy products (referred to as product codes Y01-Y25) (Supplementary Table S1) were randomly selected and analyzed in this study. Each product was purchased in a single package from drug stores, hypermarkets, and retailers in Bangkok, Thailand, and other countries. The dietary supplements (P01-P13) were in tablet and powder forms which indicated probiotics with or without quantity on the labels. The dietary supplements were stored at room temperature as recommended by the manufacturers. The dairy products were all yogurt with indicated probiotics on the labels. The yogurt products were transported in the cooling bag and stored at 4 °C as recommended by the manufacturers.

### 16 S and internal transcribed spacer (ITS) sequencing analyses

DNA was extracted from 250 µl of dietary products using DNeasy PowerSoil Pro (Qiagen). The powder of dietary supplements was suspended in 2 ml sterile PBS prior to DNA extraction.

DNA quality was determined by measuring the 260/280 nm absorbance rationo using NanoDrop spectrophotometer (Thermo Fisher Scientific). The quantity of extracted DNA was assessed using a Qubit fluorometer with a Qubit dsDNA High Sensitivity Assay kit (Thermo Fisher Scientific), which provides accurate quantitation within a detection range of 0.1–120 ng. Populations of microbiota were identified by 16 S and ITS ribosomal RNA (rRNA) sequencing. For taxonomic identification, DNA library was prepared using a Swift amplicon 16 S and ITS panel (Swift Biosciences, MI, USA). This panel amplifies all nine hypervariable regions (V1-V9) of 16 S rRNA gene for bacterial identification, and both ITS1 and ITS2 regions for fungal identification. Library concentration was quantified using TaqMan-based qPCR assay on a CFX Opus 96 Real-Time PCR system (Bio-Rad Laboratories, CA, USA). The quantification was performed based on a library size of 475 bp, and the final library pool was diluted to 2 nM.The library pool was denatured with 0.2 N NaOH and further diluted to 10 pM with pre-chilled HT1 buffer. The denatured library (600 µl) was loaded onto with a MiSeq v2 cartridge and sequenced using the MiSeq platform (Illumina). The sequencing analysis of 16 S rRNA gene and ITS regions was performed Using Illumina’s BaseSpace platform version 5.36.1. Quality control and sequence processing were performed following standard bioinformatics procedures. Raw sequence data were initially assessed using FastQC, followed by adapter trimming using Trimmomatic. Reads were filtered based on quality thresholds: Phred quality score ≥ 20, minimum read length of 50 bp after trimming, and removal of reads containing ambiguous bases. Error correction was performed using AfterQC. For taxonomic classification, sequences were clustered into Operational Taxonomic Units (OTUs) at 97% similarity threshold for 16 S rRNA data, while dynamic thresholds were applied for ITS data using the UNITE database. Chimeric sequences were removed using UCLUST. Only OTUs with a minimum abundance of 0.005% of total reads were retained for downstream analysis. Alpha and beta diversity analyses were performed using QIIME 2. For taxonomic classification, the GreenGenes database version 13_5 (last updated in May 2013) was used for 16 S rRNA gene sequence, while UNITE Fungal ITS Database v7.2 (Version 01.12.2017) was employed for ITS regions.

### Isolation of bacteria and culture conditions

Microorganisms were isolated from dietary supplements and dairy products using De Man Rogosa & Sharpe medium supplemented with 0.05% L-cysteine (MRSc)^43^. The powder of dietary supplements was suspended in 2 ml sterile phosphate buffered saline, pH 7.4 (PBS containing 0.137 M NaCl, 2.683 mM KCl, 8.101 mM Na_2_HPO_4_, 1.470 mM KH_2_PO_4_; Oxoid, Hampshire, England) and thoroughly mixed. Twenty microliters of the suspension or dairy products was inoculated into 13 ml of MRSc broth and incubated under both aerobic and anaerobic conditions overnight to recover different types of probiotics from the products. The microorganisms were sub-cultured on MRSc agar and incubated at 37^o^C for 2 days. Identification of the probiotics was performed using matrix-assisted laser desorption/ionization time-of-flight mass spectrometry (MALDI-TOF MS) as previously described^44^. Bacterial protein was extracted by formic acid method. Then, 1 µl of the solution was spotted onto a MSP-384 polished steel target plate (Bruker Daltonics, Germany). After drying in air, each spot was overlaid with 1 µl of formic acid. After drying in air, each spot will be overlaid with 1 µl of matrix, α-cyano-4-hydroxycinnamic acid (HCCA) (Bruker Daltonics, Germany) dissolved in a solution of 50% acetonitrile, 2.5% trifluoroacetic acid and 47.5% water. Each spot was measured in 200 shot steps for a total of 1200 laser shots using a MALDI-TOF Mass Spectrometer Autoflex speed (Bruker Daltonics, Germany) and FlexControl software (version 3.4.135, Bruker Daltonics, Germany). Spectra were analyzed within a mass range of 2,000–20,000 Da. Identification was achieved using the MALDI-Biotyper software (version 3.1, Bruker Daltonics, Germany). Interpretation will be performed according to the manufacturer’s recommendation. The cut-off scores were ≥ 2.00 and 1.700–1.900 for the species and genus levels, respectively.

16 S rRNA gene (for bacterial identification) or ITS regions (for fungal identification) were amplified and sequenced using the Sanger sequencing method to confirm identification of the isolated microorganisms. The probiotic bacteria and yeast were suspended in MRSc broth with 20% glycerol, except for *Streptococcus thermophilus*, which was suspended in M17 broth with 0.1% skim milk and 25% glycerol to enhance cell viability and cryoprotection efficiency, and then stored at -80 ºC for further experiments. The isolates were sub-cultured twice in the same media before performing assays.

### Probiotic growth condition and inoculum Preparation

In brief, a single colony was picked and inoculated into 2 ml of an appropriate broth medium: MRSc for all probiotics, except *S. thermophilus* was cultured in M17 with 0.1% skim milk, *Enterococcus* spp. was cultured in LB medium, and yeast was culture in yeast extract–peptone dextrose (YPD) broth. The broth culture was incubated at optimal temperature and oxygen requirements as follows: *Bifidobacterium* sp. and *L. delbrueckii* were grown in an anaerobic jar with anaerobic gas generating sachet (O_2_ < 0.1% and CO_2_ 7–15%; Oxoid) for 48 h; *Lactobacillus* spp. was grown at 37^o^C for 18 h without shaking; *S. thermophilus* was grown at 42 ºC for 18 h without shaking; *Enterococcus* spp. and yeast were grown at 37^o^C for 18 h with shaking at 200 rpm. Microbes were harvested by centrifugation at 16,2000 ×g for 5 min and the culture supernatant was discarded. The probiotic pellets were re-suspended in PBS and adjusted an optical density at 600 nm to 0.4 with approximately 1 × 10^8^ CFU/ml.

### Cell lines and culture conditions

THP-1 cells (TIB-202, American Type Culture Collection), a human monocytic cell line, were maintained in RPMI 1640 medium supplemented with 10% heat-inactivated fetal bovine serum (FBS) and 100 U/ml penicillin and 100 µg/ml streptomycin (Gibco) at 37 ºC with 5% CO_2_ for 2 days. 4 × 10^5^ cells were seeded into each well of 24-well tissue culture plate and activated with 50 ng/ml of phorbol-12-myristate-13-acetate (PMA; Sigma) for 2 days for the differentiation into macrophages. After removing PMA, the THP-1 monocyte-derived macrophages were incubated with fresh medium at 37 ºC with 5% CO_2_ for 2 days. Prior to performing the experiments, the macrophages were washed twice with 1 ml of Dulbecco’s phosphate-buffered saline (DPBS; Hyclone) and maintained in 0.5 ml of RPMI medium without FBS and antibiotics^45^.

A human NK cell line, NK-92MI (CRL-2408, American Type Culture Collection), was maintained in minimum essential medium (MEM-α) without nucleosides and supplemented with 2.2 g/l sodium bicarbonate, 2 mM L-glutamine, 100U/ml penicillin and 100 µg/ml streptomycin, 0.02 mM folic acid (Sigma-Aldrich), 0.2 mM inositol (Sigma-Aldrich), 0.1 mM 2-mercaptoethanol (Sigma-Aldrich), 12.5% fetal bovine serum (Hyclone) and 12.5% horse serum (Gibco) at 37 ºC with 5% CO_2_. Cells were subcultured every 2–3 days. The cells at a concentration of 6 × 10^5^ cells/ml for 0.5 ml without supplements and antibiotics were seeded into each well of a 24-well tissue culture plate and incubated at 37 ºC, 5% CO_2_ before performing the experiments.

### Antimicrobial activity

Probiotics from dietary supplements and dairy products were examined for antimicrobial activity against 5 pathogenic bacteria by agar overlay method as previously described^29^. The target bacteria were susceptible and resistant to several antibiotics. These included *S. aureus* ATCC25923, *S. aureus* DMST20654 (MRSA), *Escherichia coli* ATCC25922 and *E. coli* PB1(ESBL) and PB231 (CRE)^46^. In brief, the standardized inoculation method, micropipette, dispensed one microliter of probiotic suspension at a concentration of approximately 1 × 10^8^ CFU/ml onto the dry surface of MRSc agar. The plate was incubated with appropriate and consistent growth conditions for 24 h to minimize variability. The target bacteria were cultured in LB broth at 37 ºC with shaking at 200 rpm overnight and adjusted OD at 600 nm to 1.0 (approximately 10^9^ CFU/ml) before mixing 1 ml of bacterial suspension with 9 ml of 0.8% Muller-Hinton agar (MHA) to obtain a final concentration of 1 × 10^8^ CFU/ml. Ten milliliters of each pathogen in soft agar were gently overlaid on MRSc agar plates, which contained probiotic growth in a spot (5 mm diameter) on the surface. The plate was incubated at 37 ºC for 24 h. The clear zones around the probiotics spot were measured. The diameter of the inhibition zone was interpreted as follows: >20 mm as strong activity, 10–20 mm as moderate activity, < 10 mm as weak activity, and no inhibition zone as no activity. Disk diffusion of standard antibiotics: tetracycline (TET), ceftazidime/clavulanic acid (CAZ/CLA), meropenem (MEM) and vancomycin (VAN) (Oxoid, UK) were performed as positive controls.

### Phagocytosis assay

To determine whether probiotics can enhance phagocytic activity, carboxyfluorescein diacetate succinimidyl ester (CFSE), a fluorescent membrane permeable ester that activates by intracellular esterase was applied to detect phagocytosed *E. coli* ATCC25922 in THP-1 monocytes^47^. First, *E. coli* ATCC25922 was grown in 10 ml of LB broth at 37℃ with shaking at 200 rpm for 18 h. The bacteria were harvested by centrifugation in 4000 ×g at room temperature for 10 min. After discarding the supernatant, the pellet was washed twice with PBS (pH 7.4) and resuspended in PBS to an OD at 600 nm to 1.0 (approximately 10^9^ CFU/ml). The bacteria were concentrated 25 times by centrifugation and resuspended with PBS. The bacterial suspensions were serially diluted and plated on LB agar plates to enumerate the colony-forming units before inactivation. The inactivation step was applied by placing the obtained solutions in the water bath at 70 ℃ for 40 min. To ensure that all bacterial cells were killed, a cultured plate of bacteria was provided and incubated overnight^48^. Heat-killed *E. coli* was adjusted concentration to 2.5 × 10^10^ CFU/ml according to the bacteria plate count before heat-inactivation. Five microliters of the bacterial suspension then were incubated at 37 °C for 30 min in the dark with an equal volume of a 10 µM solution of CFSE (BD Horizon). The bacteria were collected, and excess dye was removed by centrifugation at 13,800 ×g for 5 min, washed twice with PBS and resuspended in 0.5 ml RPMI medium without FBS.

One hundred microliters of THP-1 monocyte cells at a concentration of 5 × 10^5^ cells/ml in RPMI were seeded into each well of a 96-well plate. The cells were stimulated with 100 µl of probiotics in RPMI at MOI of 200 at 37 ºC with 5% CO_2_ for 1 h. Culture supernatant was aspirated after centrifugation at 600 ×g for 5 min and washed twice with 200 µl of DPBS. Heat-killed *E. coli* stained with CFSE was added into each well to obtain the final concentration of *E. coli* at 5 × 10^8^ CFU/ml in a final volume of 200 µl. The plate was incubated at 37 ºC with 5% CO_2_ for 2 h. The phagocytosis was stopped by incubation on ice for 5 min. After washing twice with cold DPBS, 100 µl of cells in DPBS were mixed with 100 µl of 0.04% trypan blue quenching solution and then placed on ice for 15 min. Cells were washed twice with DPBS and resuspended in 200 µl of cold DPBS. The phagocytosis was assessed using a FACSCalibur flow cytometer (Becton Dickinson, Franklin Lakes, NJ, USA). Three independent experiments in triplicate were performed. The phagocytic index was defined as the percentage of monocyte cells containing *E. coli* stained with CFSE (% phagocytosis) multiplied by the mean fluorescence intensity of the cells.

### Macrophages stimulation with probiotics and *E. coli* lipopolysaccharide.

The THP-1 monocyte-derived macrophages were treated with probiotics at 4 × 10^6^ CFU alone or 1 µg/ml lipopolysaccharide of *E. coli* (*E. coli*-LPS) (Sigma-Aldrich) alone, or 1 µg/ml of *E. coli-*LPS together with probiotics at 4 × 10^6^ CFU^45^. Untreated macrophages in the RPMI medium were used as a negative control. Five hundred microliters of probiotic suspension at a concentration of 8 × 10^6^ CFU/ml in RPMI medium was added into the cells to obtain MOI of 10 and incubated at 37 ºC with 5% CO_2_ for 18 h. The culture supernatant was collected after centrifugation at 12,000 rpm for 5 min and stored at − 80 ºC until use. The experiments were performed once in duplicate. The concentrations of IL-1β, IL-1RA, IL-6, IL-8, IL-10, IL-12p70, and TNF-α cytokines in the supernatant were evaluated using a Milliplex^®^MAP Human High Sensitivity T cell magnetic bead panel kit (Merck KGaA, Darmstadt, Germany). The cytokine levels were analyzed using the Luminex analyzer MAGPIX^®^ system with xPOTENT^®^ software.

### Probiotics activation of NK cells

Five hundred microliters of probiotic suspension at a concentration of 6 × 10^6^ CFU/ml was added into NK-92 MI cells to obtain MOI of 10 and incubated at 37 ^o^C with 5% CO_2_ for 24 h. NK-92 MI cells treated with 20 ng/ml PMA plus 1 µg/ml ionomycin were used as a positive control. The experiments were performed in three independent experiments in triplicate. Cell culture supernatants were collected by centrifugation at 1,000 ×g for 5 min and 12,000 ×g for 5 min and stored at − 80 ^o^C until use. Cell-free supernatant was diluted 1:20 before IFN-γ measurement using a Human IFN-γ ELISA kit (BD Biosciences, Franklin Lakes, NJ, USA). The detection limit for IFN-γ assay was 4.7 pg/ml.

### Statistical analysis

All statistical data were analyzed using GraphPad Prism version 7.0 (GraphPad Software Inc, San Diego, CA, USA). Continuous data were reported as mean ± SD. Comparison between groups was analyzed using a one-way ANOVA test. The Mann-Whitney U test tested the median difference of non-normally distributed data between groups. P value < 0.05 was considered statistically significant.

## Electronic supplementary material

Below is the link to the electronic supplementary material.

Supplementary Material 1

Supplementary Material 2

## Data Availability

The metagenomic data generated during the current study is available in the SRA database under BioProject accession number PRJNA1203583. The datasets used and/or analysed during the current study are available from the corresponding author on reasonable request.
