# Supplementary material for: Metagenomic evaluation, antimicrobial activities, and immune stimulation of probiotics from dietary supplements and dairy products
Source: Sci Rep. 2025 Apr 4;15:11537. doi: 10.1038/s41598-025-95664-w (PMC11971409; doi:10.1038/s41598-025-95664-w)
Supplement: Supplementary file 1 — Supplementary Material 1 [file 41598_2025_95664_MOESM1_ESM.pdf]

Supplementary file

Metagenomic evaluation, antimicrobial activities, and immune stimulation of probiotics from dietary supplements and dairy products

Piyaorn Chornchoem[1], Sarunporn Tandhavanant[1], Natnaree Saiprom[1], Anucha Preechanukul[1,2], Nartthawee Thongchompoo[3], Insee Sensorn[3], Wasun Chantratita[3], Narisara Chantratita[1,4*]

[1]Department of Microbiology and Immunology, Faculty of Tropical Medicine, Mahidol University, Bangkok, Thailand

[2]Division of Infection and Immunity, University College London, London, UK

[3]Center for Medical Genomics, Faculty of Medicine Ramathibodi Hospital, Mahidol University, Bangkok, Thailand

[4]Mahidol-Oxford Tropical Medicine Research Unit, Faculty of Tropical Medicine, Mahidol University, Bangkok, Thailand

***Correspondence:** Narisara Chantratita, Department of Microbiology and Immunology, Faculty of Tropical Medicine, Mahidol University 420/6 Rajvithi Road, Bangkok 10400, Tel: +66819099772, E-mail: narisara@tropmedres.ac

This document provides information on 2 supplementary figures and 4 supplement data.

Supplementary figures

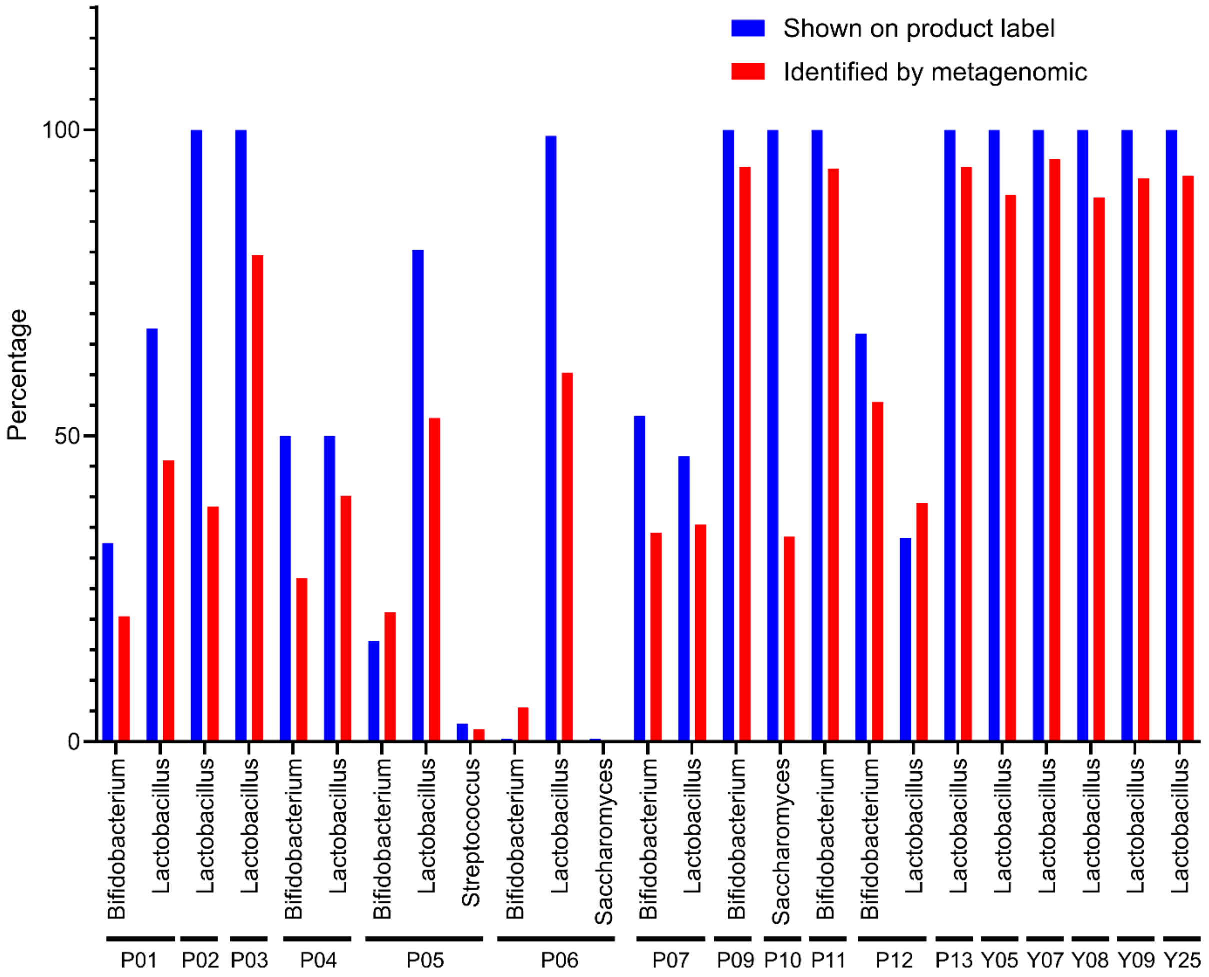

Supplementary Figure 1. Percentage of microorganisms labeled on the product (blue bar) and percentage of abundance microorganisms identified by metagenomic analysis (red bar) at the genus level.

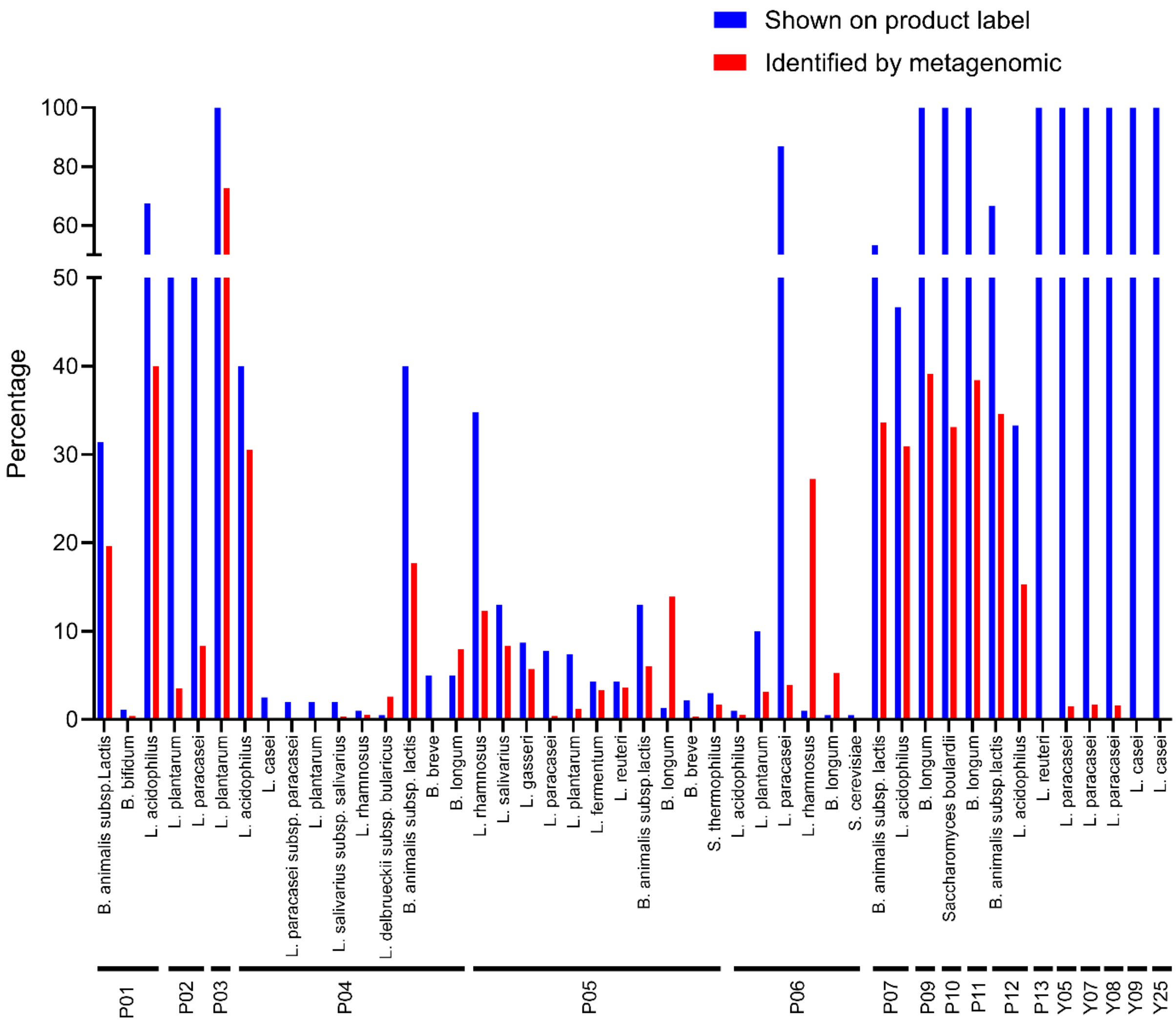

Supplementary Figure 2. Percentage of microorganisms labeled on the product (blue bar) and percentage of abundance microorganisms identified by metagenomic analysis (red bar) at the species level.

Supplementary data

Supplementary Data 1 Composition of microorganisms labeled on the product and percentage abundance of microorganisms identified by metagenomic analysis

Supplementary Data 2 Top10 microorganism classification identified by metagenomic analysis

Supplementary Data 3 List of isolated and characterized microorganisms in this study

Supplementary Data 4 Benefit of dietary supplements and dairy products claimed by manufacturers
